# Oxidative-antioxidant status of follicular fluid in IVF patients: Influence of ovarian response and oocyte-cumulus complex morphology

**DOI:** 10.5935/1518-0557.20250173

**Published:** 2026

**Authors:** Natalia Sofía Álvarez Asensio, Cecilia Haro, Ana Carolina Agüero Aguilera, Rossana Chahla, Federico Bonilla

**Affiliations:** 1 Instituto de Biología. Facultad de Bioquímica, Química y Farmacia. Universidad Nacional de Tucumán. CONICET (Consejo Nacional de Investigaciones Científicas y Técnicas). Chacabuco 461. CP 4000. San Miguel de Tucumán, Tucumán, Argentina; 2 Instituto de Bioquímica Aplicada. Facultad de Bioquímica, Química y Farmacia. Universidad Nacional de Tucumán. Balcarce 747. CP 4000. San Miguel de Tucumán, Tucumán, Argentina; 3 Centro de Medicina Reproductiva Reproducir. Virgen de las Merced 420. CP 4000. San Miguel de Tucumán, Tucumán, Argentina; 4 Instituto de Maternidad y Ginecología Nuestra Señora de las Mercedes. Sistema Provincial de Salud. Av. Mate de Luna 1535. CP 4000. San Miguel de Tucumán, Tucumán, Argentina

**Keywords:** follicular fluid, oxidative-antioxidant status, inflammatory status, oocyte-cumulus complex, in vitro fertilization

## Abstract

**Objective:**

This study assessed the oxidative-antioxidant status of follicular fluid (FF) in women undergoing *in vitro* fertilization (IVF), according to their ovarian stimulation response and the morphological characteristics of the recovered cumulus-oocyte complexes (COCs).

**Methods:**

Redox and inflammatory markers were evaluated in the FF of 77 IVF patients and 15 fertile oocyte donors.

**Results:**

Malondialdehyde and nitrite concentrations were elevated in low (LR), normal (NR) and high (HR) ovarian responders compared to controls. Superoxide dismutase (SOD) activity was higher in NR, while glutathione was increased in LR compared to controls. Glutathione peroxidase activity was similar between LR, NR, and controls, but significantly lower in HR. TNF-α was reduced across all groups, while IL-6 was significantly higher in LR and NR compared to controls. In LR, oxidative damage markers were elevated in both grade I and II COCs, along with increased SOD and glutathione, and lower TNF-α compared to controls. LR with grade I COCs showed higher catalase, glutathione, and TNF-α than those with grade II COCs. In NR, grade II COCs showed increased nitrite, catalase, and IL-6, but lower SOD and TNF-α compared to grade I COCs and controls. Embryo quality was associated with COC morphology: in LR, 71.4% of class A embryos originated from grade II COCs, whereas in NR, 81.8% of class A embryos derived from grade I COCs.

**Conclusions:**

These results highlight the dynamic interplay between redox balance, inflammation, ovarian response, and oocyte competence, emphasizing the importance of integrating biochemical and morphological markers to improve reproductive outcomes.

## INTRODUCTION

Infertility is a widespread health issue affecting individuals worldwide (Pedro *et al*., 2018). Although major advances in assisted human reproductive technologies have significantly improved the treatment of couples with reproductive problems, global infertility rates continue to rise. The etiology of infertility is diverse: 40-50% of cases are attributed to female factors, around 30% to male factors and approximately 10% to mixed causes. However, in 20-25% of couples, the cause remains unidentified, a condition referred to as unexplained infertility (Thurston *et al*., 2019; Yalçinkaya *et al*., 2013).

The success rate of *in vitro* fertilization and embryo transfer depends on several factors, including maternal age, the underlying cause of infertility, embryo quality, and lifestyle factors. Follicular fluid (FF) provides the biological microenvironment essential for oocyte development. It contains a complex mixture of steroids, metabolites, polysaccharides, proteins and various mediators, such as growth factors, reactive oxygen species (ROS) and antioxidants, which together contribute to proper follicular growth and oocyte maturation (Elizur *et al*., 2014; Freitas *et al*., 2017). ROS, molecules that are inevitably produced during cellular metabolism, have been shown to play multiple roles in female reproduction. ROS are involved as second messengers capable of modulating the expression of genes that control physiological processes in gametes and embryos (Dröge, 2002) and have been suggested to be involved in the ovarian ageing process (Debbarh *et al*., 2021). Oxidative stress, which results from an imbalance between ROS generation and antioxidant defences, can damage important cellular components such as lipids, proteins, carbohydrates and DNA (Nishihara *et al*., 2018). Moreover, ROS can trigger an inflammatory response by promoting the release of pro-inflammatory cytokines. These immune-related alterations may impact the oocyte-granulosa cell complex, disrupting the immune balance and potentially contributing to female infertility (Prins *et al*., 2020).

A delicate balance between antioxidants and oxidants is necessary for cell survival and is important for the maintenance of reproductive health of gametes. The impact of ROS on oocyte quality, implantation process and early embryonic development remains a topic of debate (Nuñez-Calonge *et al*., 2016; Prasad *et al*., 2016; Terao *et al*., 2019).

To all these reproductive health conditions, we must add the delay in motherhood as an adverse factor that makes fertility even more difficult. Currently, more and more women are postponing motherhood until later in life, thus limiting their reproductive capacity. The progressive reduction in the number of primordial follicles leads to an exponential decline in ovarian reserve from the age of 37 (Devesa *et al*., 2018). This not only reduces the number of oocytes produced, but also reduces oocyte quality (Kasaven *et al*., 2022).

The oxidant/antioxidant imbalance negatively affects all stages of reproduction and has been linked to female infertility. However, the role of ROS/antioxidants in reproductive pathophysiology is still under investigation (Debbarh *et al*., 2021; Oyawoye *et al*., 2003; Pasqualotto *et al*., 2004). The aim of this study was to evaluate the oxidative-antioxidant status of follicular fluid in patients undergoing IVF treatment, considering their ovarian response and the morphological characteristics of the retrieved cumulus-oocyte complexes.

## MATERIALS AND METHODS

This prospective study was conducted in the Fertility Unit of the Instituto de Maternidad y Ginecología Nuestra Señora de las Mercedes and Reproducir Center, Tucumán, Argentina, during the period January 2019 to December 2022. This study was approved by Bioethics Committee of the Facultad de Medicina-UNT N° 1078/2018, Tucumán, Argentina and it was conducted in accordance with the Helsinki Declaration.

### Study population

A total of 79 couples attended the Fertility Unit of the Instituto de Maternidad for *in vitro* fertilization (IVF) treatment. FF samples were analyzed from 77 women, as two had no oocytes retrieved and were therefore excluded. The mean age of the women included in the study was 34.8 years (range 23-44).

The control group included 15 healthy fertile oocyte donors, aged was 24.5 years (range 18-31). All had a normal physical and gynaecological examination, no family history of hereditary or chromosomal diseases, normal karyotype and negative screening for sexually transmitted diseases.

All subjects had provided written signed consent. This study excluded patients with polycystic ovary syndrome, with endometrioma, endocrinopathies such as untreated hypothyroidism, with metabolic syndrome, autoimmune diseases and smokers.

### Controlled ovarian hyperstimulation and collection of fluid follicular

All patients underwent controlled ovarian stimulation using a fixed-dose gonadotropin protocol (human menopausal gonadotropin, hMG 225 - 300 UI/day). The dose was adjusted according to ultrasound monitoring and estradiol measurements during stimulation follow-up.

The endogenous luteinizing hormone surge was suppressed with a GnRH antagonist protocol (Cetrotide 0.25mg/day, Cetrorelix, Merck Serono, UK). When at least three follicles reached a diameter of ≥18-22mm, final oocyte maturation was induced with recombinant human chorionic gonadotropin (r-hCG, Ovidrel^®^, 250µg subcutaneous, Merck Serono, UK). Oocyte retrieval was performed 36 hours later by transvaginal ultrasound-guide aspiration. Retrieved oocytes were separated from the FF and utilized for the IVF procedure. Oocyte donors underwent the same protocol for ovarian stimulation.

The FF contaminated with blood were excluded. The FF was centrifuged, the supernatant was aliquoted and stored at -20°C to later determine parameters.

The patients were divided into three groups according to their response to ovarian stimulation: 28 women with a low response to ovarian stimulation (LR), where the number of oocytes retrieved in each patient was between 1 and 3; the second group included 36 patients with a normal response (NR), where the number of oocytes retrieved was between 4 and 8; and the third group consisted of 13 patients with a high response (HR), where the number of oocytes retrieved in each patient was greater than 8.

### Morphological classification of cumulus-oocyte complexes and embryo quality

**Figure 1:**
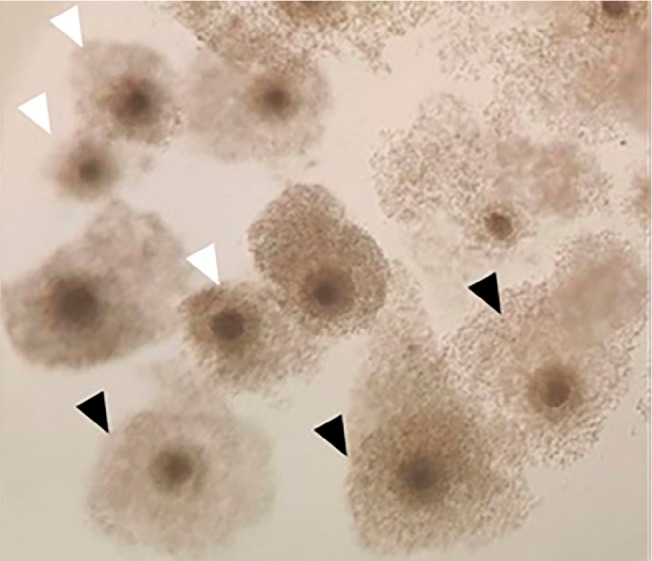
Morphological classification of cumulus-oocyte complexes. Grade I COCs are indicated with white arrows and grade II COCs with black arrows.

The recovered cumulus-oocyte complexes were classified according to their morphological aspect: compacted COC (grade I) when the oocyte was surrounded by 1 to 6 layers of tightly packed cells, and expanded COC (grade II) when the oocyte was surrounded by more than 6 layers of expanded and loose follicular cells (Figure 1).

For embryo quality assessment, the criteria of the Istanbul consensus of clinical embryologists were used as a reference framework (Alpha Scientists in Reproductive Medicine & ESHRE Special Interest Group of Embryology, 2011). The criteria included the assessment of the degree of fragmentation and blastomere symmetry on the third day after fertilization. We considered that in class A an embryo would have 8 equally sized mononucleated blastomeres, with <10% fragmentation; class B when the fragmentation was 10-20% with moderate asymmetry blastomeres; and class C when the fragmentation was > 20% with high asymmetry blastomeres.

### Oxidative stress markers

Lipid peroxidation in FF was determined by estimating malondialdehyde (MDA) as thiobarbituric acid reactive substances through the method described by Buege and Aust modificated (Buege & Aust, 1978; Yonny *et al*., 2016). MDA was measured spectrophotometrically at 535 nm under acidic conditions solution (15% trichloroacetic acid, 0.375% thiobarbituric acid and 0.25 N HCl) and subsequent alkaline hydrolysis (3 N NaOH) in 200 µL of FF samples. The results were expressed in µmol/L.

Nitrite concentration (NO_2_^-^) was measured by the Griess method modificated (Miranda *et al*., 2001). The nitrites present in the FF sample react with Griess reagent [sulfanilamide 1% (w/v), N-1-(naphthyl) ethylenediamine 0.1% (w/v) and concentrated phosphoric acid] to form a chromophore, which is measured spectrophotometrically at 545 nm, as the final product of the diazotization. The NO_2_^-^ concentration of the FF sample was determined by 1 mM Sodium Nitrite a standard curve. NO_2_^-^ concentrations were expressed in µmol/L.

### Antioxidant defences

The antioxidant status was evaluated by determining enzymatic defences: catalase (CAT), superoxide dismutase (SOD) and glutathione peroxidase (GPx) and non-enzymatic ones: glutathione (GSH).

CAT activity was measured in 10 µL FF by the Aebi method (Aebi, 1984). The spectrophotometric assay involved the decomposition of hydrogen peroxide by CAT enzyme in phosphate buffer (50 mM, pH 7) with 10 mM of hydrogen peroxide. The reaction was monitored continuously at 240 nm. Catalase activity was expressed as pmol of H_2_O_2_ /mg of protein.

SOD activity was determined by the method of Misra and Fridovich (Misra & Fridovich, 1972). SOD can inhibit the autoxidation of adrenaline in alkaline medium (glycine 50 mM, pH=10.2; adrenaline 60 mM, pH=2, Sigma Aldriech), producing an adrenochrome which was detected spectrophotometrically at 480 nm at 30°C. One unit of SOD was defined as the amount of enzyme that inhibited adrenochrome formation by 50%. Enzyme activity was expressed in mIU/mg of protein.

Glutathione peroxidase (GPX) catalyses the reduction of organic and inorganic hydroperoxides using reduced glutathione as an electron donor. The activity of this enzyme was determined by the method of Flohé and Günzle (Flohé & Günzler, 1984). The remaining GSH reacts with 5,5’-dithiobis-(2-nitrobenzoic) acid (DTNB) to form a yellow complex, which was detected spectrophotometrically at 412 nm. A calibration curve was established using 40 mM GSH to determine the concentration of the remaining GSH. The reagents used were 0.1 M phosphate buffer (pH 7.4), 4 mM GSH, DTNB 10 mM and 5% sulfosalicylic acid. GPx activity was expressed in µmol/mg protein.

The concentration of GSH, a non-enzymatic antioxidant, was determined by the method described by Weckbecker and Cory (Weckbecker & Cory, 1988), which is based on the reduction of DTNB (Ellman’s reagent) by glutathione (-SH) groups and the formation of a coloured compound which is detected spectrophotometrically at 412 nm. The concentration of GSH was expressed in µmol/mg protein determined from a standard curve constructed with known concentrations of GSH.

### Proinflammatory cytokines

IL-6 and TNF-α concentration were determined with the BD OptEIA™ ELISA kit (BD Biosciences, US) following the manufacturer’s recommendations. Concentrations were expressed in pg/mL.

### Protein determination

FF protein was measured by the colorimetric method of Biuret using Proti 2 commercial reagent (Wiener lab.) according to manufactured recommendation. Measurements were performed at 540 nm. Results were expressed in mg/mL.

### Statistical analysis

Statistical analyses were performed using InfoStat V.2020 statistical software. The data are reported as median and quartiles (Q_1_-Q_3_). The comparative study was performed using the non-parametric Kruskal-Wallis test. A significance level of *p*<0.05 was adopted.

## RESULTS

A total of 77 FF samples were analyzed from women undergoing IVF treatment, classified into three groups according to their response to ovarian stimulation: LR, NR, and HR. The biochemical characteristics of these groups are presented in Table 1. No significant differences were observed in terms of BMI and baseline hormone levels between the groups. However, the control group participants were significantly younger than those in the LR, NR and HR groups.

**Table 1:** Biochemical characteristics in different patient groups.

	LR (n=28)	NR (n=36)	HR (n=13)
**Age (years)**	37.0 (32.0-39.0)	33.0 (30.0-39.0)	35.0 (31.0-38.0)
**BMI (kg/cm^2^)**	29.5 (25.0-31.0)	28.9 (26.0-30.0)	28.7 (28.3-34.0)
**FSH (UI/L)**	9.0 (6.0-14.0)	7.4 (6.0-8.5)	6.9 (5.7-7.5)
**LH (UI/L)**	6.0 (3.0-8.0)	5.6 (4.2-8.0)	5.2 (4.0-7.4)
**E2 (pg/mL)**	44.0 (33.0-62.9)	38.0 (29.0-59.0)	34.0 (28.0-56.0)
**PRL (µg/L)**	18.0 (13.0-23.0)	16.0 (12.5-24.0)	19.0 (14.0-23.0)
**TSH (mU/L)**	1.6 (1.2-2.1)	2.0 (1.2-2.5)	1.8 (0.9-2.1)
**T4 free (ng/dL)**	1.3 (1.2-1.6)	1.3 (1.2-1.5)	1.3 (1.0-1.7)
**Glucose (mg/dL)**	93.0 (91.0-96.0)	90.0 (82.0-96.0)	88.5 (83.0-91.0)
**Insulin (U/mL)**	10.0 (7.0-14.0)	11.0 (9.0-17.0)	7.0 (6.0-8.0)
**HOMA**	2.4 (1.3-3.0)	2.4 (1.9-4.0)	1.6 (1.5-2.5)

LR: Lower responder; NR: Normal responder; HR: High responder; BMI: body mass index; FSH: Follicle-Stimulating Hormone; LH: luteinizing hormone; E2: estrogen; PRL: prolactin; TSH: Thyroid-stimulating hormone; HOMA: Homeostatic Model Assessment.

### Oxidative and antioxidant markers

**Figure 2:**
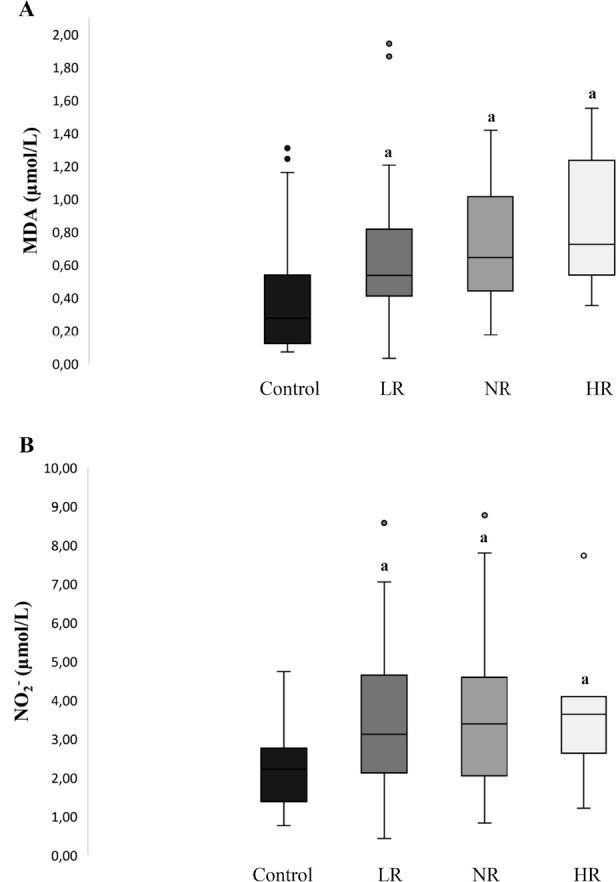
Oxidative stress markers. A) MDA: malondialdehyde, B) Nitrites (NO_2_-). LR: Lower responder; NR: Normal responder; HR: High responder. ^a^ Significantly different from control p<0.05.

The concentration of MDA, one of the key indicators of lipid peroxidation, were significantly higher in all three patient groups studied compared to the controls (Figure 2A). Similarly, NO_2_^-^ concentration, another marker of oxidative stress, were elevated in all three study groups compared to the controls (Figure 2B).

The antioxidant defences evaluated in the different groups are summarized in Table 2. All 3 patient groups showed CAT enzyme activity similar between groups. SOD activity was notably higher in the NR group, while GSH levels were increased in the LR group compared to controls. GPx levels in the LR and NR groups were similar to control, while the HR women showed a significant lower GPx activity than the other groups studied.

**Table 2:** Antioxidants defences in different groups.

	LR (n=28)	NR (n=36)	HR (n=13)	Control (n=15)
Age (years)	37 (32-39)^*^	33 (30-39)^*^	35 (31-38)^*^	24 (22-27)
CAT (pmol/mg proteins)	1.0 (0.6-1.6)	1.4 (0.8-2.2)	0.7 (0.6-1.2)	1.3 (0.6-2.1)
SOD (mU SOD/mg proteins)	296.4 (129.0-451.6)	340.0 (204.7-496.6)^*^Ϯ	238.6 (170.4-292.3)	200.0 (105.7-246.7)
GPX (µmol/mg proteins)	24.1 (12.7-39.5)	26.5 (17.0-38.5)	11.4 (7.8-15.4)^*^Ϯ	26.7 (21.4-51.4)
GSH (µmol/mg proteins)	34.5 (16.7-68.9)^*^Ϯ	17.2 (13.6-37.5)	17.3 (9.9-46.2)	13.9 (7.3-21.9)

LR: Lower responder; NR: Normal responder; HR: High responder; CAT: catalase; SOD: Superoxide dismutase; GPx: glutathione peroxidase; GSH: glutathione. Data are presented as median (quartile1-quartile3).

* Significantly different *p*<0.001 respect to control group. Ϯ Significantly different *p*<0.001 respect to the other groups.

### Inflammatory markers

**Figure 3:**
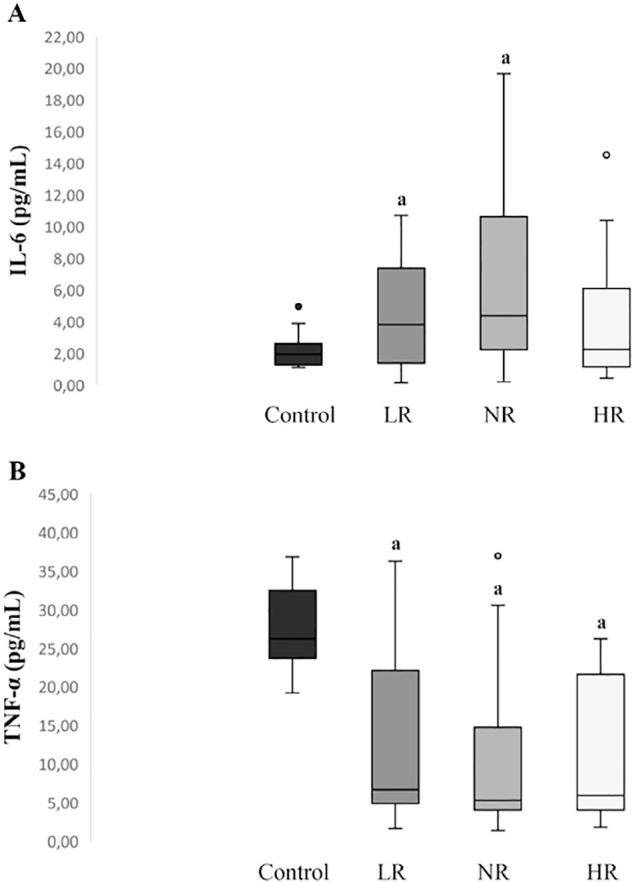
Proinflammatory cytokines in FF. A) IL-6: interleukin 6. B) TNF-α: tumor necrosis factor alpha. LR: Lower responder; NR: Normal responder; HR: High responder ^a^ Significantly different from control *p*<0.05.

Concentration of TNF-α and IL-6 cytokines in FF were evaluated as inflammatory markers. TNF-α concentrations were consistently lower in the three patient’s groups than in controls (Figure 3B). On the other hand, IL-6 levels were significantly elevated in the LR and NR women compared to the control group (Figure 3A).

### Oxidative-inflammatory state and COC morphological grading

**Figure 4:**
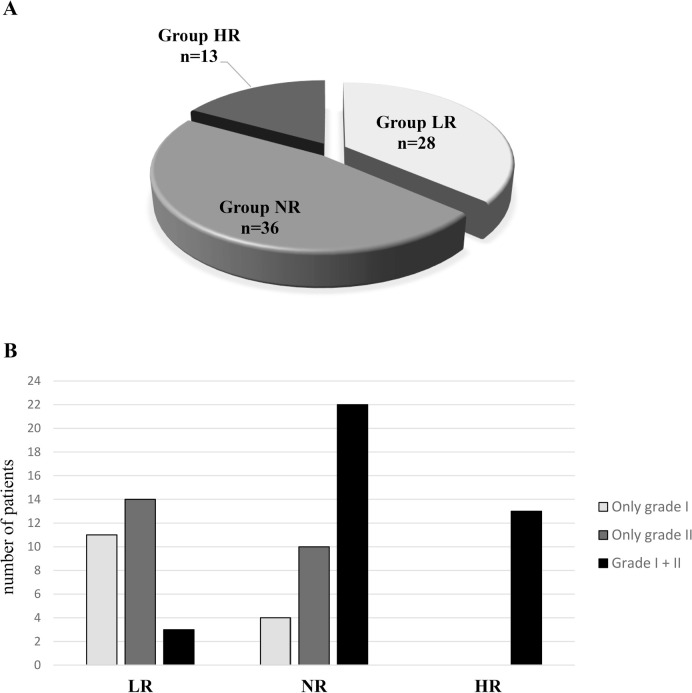
Patients undergoing IVF treatment. A) Groups according to response to ovarian stimulation, B) Morphological classification of COCs recovered in different groups of patients. LR: Lower responder; NR: Normal responder; HR: High responder; COCs: cumulus-oocyte complexes.

Considering that the morphological characteristics of the COC serve as the first observable indicator during oocyte retrieval in IVF and may reflect the oxidative-inflammatory conditions of the FF, we examined the oxidative-inflammatory profile of FF according to the classification of COCs into grade I and grade II across the different study groups. In the LR group, 11 women exclusively presented grade I COC, 14 displayed grade II COC, and 3 exhibited a mix of both grades. In the NR group, 22 women presented both grade I and II COC, 10 had only grade II COC, and 4 had exclusively grade I COC (Figure 4A). All HR women presented a combination of grade I and II COC. The oxidative-inflammatory parameters in the FF were analysed only in cases where women had either grade I or grade II COCs, without a mix of grades (Figure 4B).

**Figure 5:**
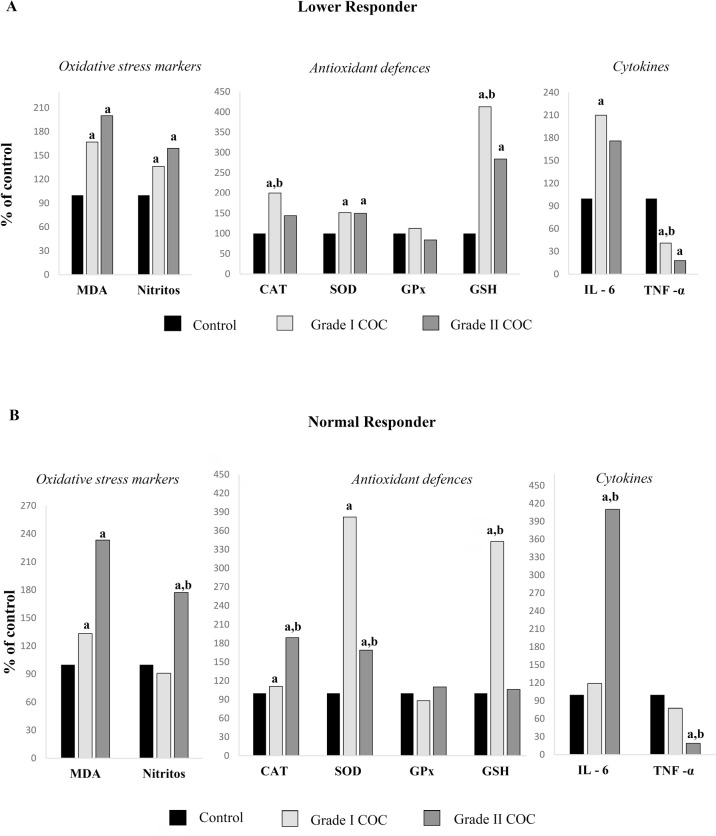
Redox and inflammatory profile in LR and NR women according COCs morphological grading. The results are expressed in percentage (%) of the values obtained in women LR and NR versus controls, considering the values in controls as 100%. LR: Lower responder; NR: Normal responder; COCs: cumulus-oocyte complexes. ^a^ Significantly different *p*<0.001 respect to control group. ^b^ Significantly different *p*<0.001 respect to the other groups.

In the LR women, both grade I and II COC groups exhibited similar levels of oxidative damage, indicated by elevated MDA and NO_2_^-^, accompanied by an increased SOD activity and GSH, along with lower TNF-α concentrations compared to control group (Figure 5A). However, LR women with grade I COC showed higher levels of CAT, GSH and TNF-α compared to those with grade II COC.

In the NR group, only women with grade II COC showed higher levels of NO_2_^-^ compared to control and grade I COC groups, respectively (Figure 5B). In addition, NR women with grade II COCs showed significantly higher CAT activity, along with lower levels of SOD and GSH compared to those with grade I COCs. Also, they had a higher concentration of IL-6 and a lower concentration of TNF-α compared to grade I COC (*p*<0,01).

### Fertilization and embryo quality

The maturity of the oocytes retrieved, fertilization rate and 3rd day embryo count were similar between grade II and I COCs in the groups LR women and NR (Table 3). However, in LR women, 71.4 % of class A embryos originated from grade II COCs, whereas in NR women, 81.8% of class A embryos were derived from grade I COCs.

**Table 3:** Fertilization and embryo quality outcomes.

	Grade COC	Oocyte maturity (%)	Fertilization rate (%)	Embryo day 3 (%)	Class A embryos (%)
**LR**	**Grade I** **(n=19)**	13/19 (68.4)	13/13 (100)	13/13 (100)	6/13 (46.1)
	**Grade II** **(n=27)**	24/27 (88.9)	22/24 (91.7)	21/22 (95.5)	15/21 (71.4)
**NR**	**Grade I** **(n=17)**	15/17 (88.2)	13/15 (76.5)	11/13 (84.6)	9/11 (81.8)
	**Grade II** **(n=55)**	46/55 (83.6)	42/46 (76.4)	38/42 (90.5)	21/38 (55.3)

LR: Lower responder; NR: Normal responder; COCs: cumulus-oocyte complexes.

## DISCUSSION

FF provides a vital microenvironment for oocyte development, containing a complex mix of steroids, metabolites, proteins, and signalling molecules, including ROS and antioxidants. The redox balance in FF is essential for proper follicular growth and oocyte maturation (Chen *et al*., 2023). Disruptions in this balance could impair oocyte quality, potentially affecting ovarian stimulation outcomes and lowering reproductive success.

Our results showed increased levels of MDA and NO₂⁻ in the FF of all IVF patient groups (LR, NR, and HR) compared to controls. This indicates enhanced reactive species generation in women undergoing IVF treatment, regardless of the type of ovarian response. These findings may reflect an age-related change in the oxidant/antioxidant balance, as the control group (oocyte donors), consisting of younger women who also underwent ovarian stimulation, did not exhibit elevated reactive species levels. The absence of significant variations in oxidative stress markers between patient groups may suggest that the redox imbalance is not primarily due to the magnitude of the ovarian response, but rather to intrinsic patient-related factors such as age, basal metabolic state or subclinical inflammation. This suggests that redox homeostasis may be better preserved in younger ovaries, possibly due to more efficient antioxidant systems and a follicular microenvironment less affected by ageing changes. Such preservation could help maintain a balanced oxidative state despite the hormonal challenge of ovarian stimulation, underscoring the relevance of age as a critical determinant in the regulation of the follicular redox environment. Previous studies have reported a significant increase in lipoperoxidation level in the FF from women aged 37 years and older, indicating a decline in ROS scavenging efficiency with advancing age (Debbarh *et al*., 2021). These age-related changes may be due to alterations in the ovarian microenvironment, contributing to an imbalance in oxidant/antioxidant dynamics. Consistent with our findings, Nuñez-Calogne *et al*. observed elevated MDA levels in LR compared to oocyte donors, along with higher NO₂⁻ concentrations in both low and high responder (Nuñez-Calonge *et al*., 2016). In contrast, another study reported that HR and NR exhibited similar oxidative stress levels, whereas LR showed significantly elevated MDA concentrations (Uppangala *et al*., 2020). These contrasting results highlight the variability in oxidative stress profiles across different patient groups, underscoring the complexity of redox balance regulation within the follicular microenvironment and its potential dependence on individual patient characteristics and ovarian response type.

The pattern of antioxidant defences in FF varied according to the type of ovarian response in our study, influencing the reactive species scavenging efficiency. Interestingly, NR women showed higher levels of SOD, while LR demonstrated elevated GSH concentrations, likely as a compensatory mechanism for increase in reactive species. Conversely, HR exhibited reduced levels of GPx compared to the other groups. One possible explanation is that the increased metabolic demand associated with the simultaneous development of multiple follicles could lead to excessive ROS generation in FF, resulting in increased GPx utilization. Alternatively, the lower GPx activity observed might reflect a subtle dysregulation in antioxidant enzyme expression or function, possibly influenced by the altered endocrine milieu or the accelerated follicular dynamics characteristic of hyper-responsiveness to stimulation. These mechanisms are not necessarily mutually exclusive and may act in parallel, shaping a follicular environment that, although favorable in quantitative terms, may be suboptimal for maintaining redox balance. These observations highlight the relevance of evaluating not only the number of oocytes retrieved, but also the biochemical conditions of the follicular environment. Similar findings had been reported, with variations in antioxidants profile associated with ovarian response type in patients undergoing IVF (Nuñez-Calonge *et al*., 2016; Thaker *et al*., 2020). However, unlike our results, Nuñez-Calogne *et al*. detected lower GPx activity in LR patients (Nuñez-Calonge *et al*., 2016).

Regarding inflammatory markers, we detected higher IL-6 levels in the FF of LR and NR women compared to HR and controls. In line with our findings, other authors also reported elevated IL-6 concentrations in LR patients, while HR women exhibited levels similar to those in the control group (Nuñez-Calonge *et al*., 2016). Similarly, Taghavi *et al*. found increased IL-6 levels in women with poor ovarian response compared to those with normal ovarian function (Taghavi *et al*., 2014). IL-6 is a cytokine pleiotropic that plays an important role in folliculogenesis, regulating processes such as cell proliferation, differentiation, follicle survival, atresia, and oocyte maturation (Field *et al*., 2014). Low FF IL-6 concentrations have been associated with the retrieval of more mature oocytes (Ilhan *et al*., 2023), which might suggest that the lower IL-6 levels observed in our control group reflect a follicular environment more conducive to oocyte maturation.

In addition, in our study, TNF-α concentrations were consistently lower in the patient groups compared to the control group, which comprised young oocyte donors without fertility issues. This observation highlights a potential difference in the inflammatory microenvironment during follicular development between the donors and patients undergoing IVF. Interestingly, these findings contrast with those of Huang *et al*., who observed significantly elevated FF TNF-α levels in patients with diminished ovarian reserve compared to those with normal ovarian reserve (NOR) (Huang *et al*., 2023). Moreover, the TNF-α levels in their NOR group were notably higher than those observed in our control group. These discrepancies may be attributed to differences in the composition of the control population. While Huang *et al*. defined their NOR group as infertile women undergoing IVF due to male, tubal or endometrial factors, our control group consisted exclusively of healthy, young oocyte donors, representing a baseline of optimal reproductive conditions (Huang *et al*., 2023). TNF-α is locally produced in the ovary by several cell types, including macrophages, oocytes, corpora lutea, theca, and granulosa cells, and plays a pivotal role in modulating angiogenesis throughout the luteal phase. It is also a key regulator in the growth and selection of antral follicles, with its local concentration likely influencing the follicular response. TNF-α signalsdistinct receptors: TNF-α-R1, primarily associated with the transduction of death signals, and TNF-α-R2, which is mainly involve in promoting cell survival and proliferation (Alhilali *et al*., 2020; Field *et al*., 2014). Thus, the net biological effect of TNF-α within the follicular environment may depend not only on its concentration but also on the relative expression of its receptors, an expression profile that may shift with age or under different pathophysiological conditions. In young, reproductively competent women, a predominance of TNFR2-mediated signaling may support granulosa cell function and oocyte maturation, whereas in older individuals, a shift toward TNFR1 activation could contribute to increased follicular atresia. Taken together, our results suggest that the reduced TNF-α levels observed in infertile patients may not solely reflect underlying ovarian pathology, but could also indicate age-related changes in cytokine regulation and follicular signaling dynamics. In contrast, the elevated TNF-α levels detected in the donor group may serve as a marker of a more favorable follicular environment, characterized by coordinated immune-endocrine activity and enhanced reproductive competence.

The morphological characteristics of COCs represent the first insights obtained following follicular aspiration during IVF procedures. These features, such as granulosa cell mass, oocyte maturation stage and oocyte morphology, are predictive of oocyte developmental potential and fertilization success. In our study, similar maturation rates, fertilization outcomes, and embryo counts at day 3 were observed between COC grades I and II in the LR and NR groups. These finding align with those of Thanaboonyawat *et al*., who reported comparable maturation, fertilization, or cleavage rates in different COC morphologies in FSH + LH receptor-primed cycles, although compacted COCs were associated with a higher proportion of top-quality embryos (Thanaboonyawat *et al*., 2016). Interestingly, our results reveal distinct trends within specific patient groups. In LR women, the highest number of class A embryos (71%) originated from grade II COC, characterized by more than 6 layers of expanded and loose follicular cells. In contrast, in the NR group, grade I COCs, surrounded by 1 to 6 layers of tightly packed granulosa cells, produced the majority (81.8%) of class A embryos. These differences may be partly attributed to variations in the redox and inflammatory states of the follicular microenvironment. In particular, the elevated CAT and IL-6 levels observed in grade I COCs of LR women and grade II COCs of NR women may influence the production of class A embryos. CAT, a key enzyme in oxidative stress regulation, catalyses the decomposition of hydrogen peroxide into water, working synergistically with SOD as part of the first-line antioxidant defences (Ighodaro & Akinloye, 2018; Misra & Fridovich, 1972). Increased CAT activity might reflect an imbalance in the antioxidant system, potentially disrupting the redox homeostasis essential for optimal oocyte development.

Regarding IL-6, its influence on follicular dynamics may support these findings. Elevated IL-6 levels could reduce intrafollicular aromatase activity, potentially leading to decreased estradiol concentrations, an essential factor for follicular development, oocyte maturation, and fertilization potential (Pellicer *et al*., 1999). Thus, the higher IL-6 levels in specific COC grades might create a suboptimal microenvironment, adversely affecting oocyte quality and embryo development. These findings highlight that COC morphology alone may not be a reliable predictor of intrinsic oocyte competence. Instead, they emphasize the complex interplay between redox and inflammatory states, COC characteristics, and embryo quality, modulated by ovarian response and patient-specific factors.

To our knowledge, this study is the first to evaluate the redox and inflammatory profile in FF in relation to ovarian stimulation response and their association with the morphological characteristics of the recovered COCs. By focusing exclusively on grade I and II COCs, we were able to delineate the variability in oxidative-inflammatory profiles across these morphologically distinct groups. This approach provided new insights into the dynamic interplay between follicular oxidative stress, inflammatory markers, and COC quality associated with different ovarian responses. One limitation of our study was that all HR patients exhibited a mixture of grade I and II COCs, which precluded the assessment of redox and inflammatory profiles based on COC morphology. Despite this, our results reveal that the oxidative status of FF varies in a complex, non-linear manner depending on ovarian response, COC quality, and possibly ovarian ageing. This underscores the complex and dynamic interplay between redox balance, inflammation mediators, and oocyte competence. Integrating biochemical and morphological markers may provide a more accurate assessment of the follicular environment and its influence on reproductive outcomes. Further studies with larger and well-characterized cohorts are needed to elucidate these mechanisms.

## CONCLUSION

These findings provide novel evidence linking the redox and inflammatory profiles of follicular fluid to ovarian response and COC morphology in women undergoing IVF. Such insights may help refine the evaluation of oocyte quality and support the development of more targeted strategies to improve assisted reproductive outcomes.
